# Characterization of the selective in vitro and in vivo binding properties of crenezumab to oligomeric Aβ

**DOI:** 10.1186/s13195-019-0553-5

**Published:** 2019-12-01

**Authors:** William J. Meilandt, Janice A. Maloney, Jose Imperio, Guita Lalehzadeh, Tim Earr, Susan Crowell, Travis W. Bainbridge, Yanmei Lu, James A. Ernst, Reina N. Fuji, Jasvinder K. Atwal

**Affiliations:** 1 Department of Neuroscience, Genentech, Inc., 1 DNA Way, South San Francisco, CA 94080 USA; 2 Department of Preclinical and Translational Pharmacokinetics/Pharmacodynamics, Genentech, Inc., 1 DNA Way, South San Francisco, CA USA; 3 Department of Protein Chemistry, Genentech, Inc., 1 DNA Way, South San Francisco, CA USA; 4 Department of Biochemical and Cellular Pharmacology, Genentech, Inc., 1 DNA Way, South San Francisco, CA USA; 5 Department of Safety Assessment Pathology, Genentech, Inc., 1 DNA Way, South San Francisco, CA USA

**Keywords:** Crenezumab, Amyloid β, Alzheimer’s disease, Oligomeric, Mossy fiber, Vascular amyloid

## Abstract

**Background:**

Accumulation of amyloid β (Aβ) in the brain is proposed as a cause of Alzheimer’s disease (AD), with Aβ oligomers hypothesized to be the primary mediators of neurotoxicity. Crenezumab is a humanized immunoglobulin G4 monoclonal antibody that has been shown to bind to synthetic monomeric and aggregated Aβ in vitro; however, less is known about the binding characteristic in vivo. In this study, we evaluated the binding patterns of crenezumab to synthetic and native forms of Aβ both in vitro and in vivo.

**Methods:**

Crenezumab was used to immunoprecipitate Aβ from synthetic Aβ preparations or brain homogenates from a PS2APP mouse model of AD to determine the forms of Aβ that crenezumab interacts with. Following systemic dosing in PS2APP or nontransgenic control mice, immunohistochemistry was used to localize crenezumab and assess its relative distribution in the brain, compared with amyloid plaques and markers of neuritic dystrophies (BACE1; LAMP1). Pharmacodynamic correlations were performed to investigate the relationship between peripheral and central target engagement.

**Results:**

In vitro, crenezumab immunoprecipitated Aβ oligomers from both synthetic Aβ preparations and endogenous brain homogenates from PS2APP mice. In vivo studies in the PS2APP mouse showed that crenezumab localizes to regions surrounding the periphery of amyloid plaques in addition to the hippocampal mossy fibers. These regions around the plaques are reported to be enriched in oligomeric Aβ, actively incorporate soluble Aβ, and contribute to Aβ-induced neurotoxicity and axonal dystrophy. In addition, crenezumab did not appear to bind to the dense core region of plaques or vascular amyloid.

**Conclusions:**

Crenezumab binds to multiple forms of amyloid β (Aβ), particularly oligomeric forms, and localizes to brain areas rich in Aβ oligomers, including the halo around plaques and hippocampal mossy fibers, but not to vascular Aβ. These insights highlight a unique mechanism of action for crenezumab of engaging Aβ oligomers.

## Background

Alzheimer’s disease (AD) is a progressive, fatal neurodegenerative disease that develops along a continuum, culminating in neuronal atrophy and dementia. The AD brain is characterized by a number of histopathologic hallmarks, including the deposition of amyloid plaques, which are composed primarily of amyloid β (Aβ) peptides [1]. Aβ peptides can exist in multiple conformations, including soluble monomers, aggregated soluble oligomers, and insoluble fibrils [1]. While the extent to which different Aβ species contribute to the pathophysiology of AD remains uncertain, in vitro and ex vivo evidence suggests that soluble low-*n* molecular weight oligomers (including dimers and trimers, up to dodecamers) may be a major driver of neurotoxicity [2–7]. Furthermore, soluble Aβ oligomers are thought to concentrate around the dense core of plaques, generating a neurotoxic halo that contributes to local neuritic dystrophy, synaptic loss, and neurodegeneration [8, 9].

Crenezumab is a humanized immunoglobulin (Ig) isotype G4 (hIgG4) monoclonal antibody (mAb) that binds to soluble forms of synthetic Aβ, including monomers, oligomers, and fibrils, and has an ≈ 10-fold higher affinity for soluble oligomeric Aβ than for monomeric Aβ (moAβ) (0.4–0.6 vs 3.0–5.0 nM [10, 11]). In vitro, crenezumab has been shown to block Aβ aggregation, promote oligomer disaggregation, and protect neurons from oligomer-induced toxicity [11]. The IgG4 backbone also confers reduced activation of Fcγ receptors (FcγRs) compared with an IgG1 backbone and limits FcγR-mediated inflammatory activation of microglia while largely preserving FcγR-mediated microglial phagocytosis of oligomers in vitro [11]. Crenezumab’s reduced effector function may lower the risk of localized microvascular damage [12], and a safety finding that has been observed as amyloid-related imaging abnormalities (ARIA) representing vasogenic edema (ARIA-E) in clinical trials with other anti-Aβ mAbs on an IgG1 backbone [13–17].

The objectives of this study were to investigate the in vitro and in vivo binding characteristics of crenezumab to various forms of Aβ to gain a better understanding of target engagement in the brain and further elucidate crenezumab’s mechanism of action.

## Materials and methods

### Mice

All in vivo binding studies used 6- to 12-month-old plaque-bearing male and/or female PS2APP mice on a homozygous C57BL/6 background [18, 19]. PS2APP mice co-express human APP (hAPP) with the Swedish mutation K670N/M671L and human presenilin 2 with the N141I mutation, driven by Thy1 and PrP promoters, respectively. PS2APP-green fluorescent protein (GFP) mice were generated by crossing the PS2APP mice with the Thy1_GFP M-line—a previously characterized GFP reporter line that expresses GFP in a subset of neurons [20]. PS2APP mice were crossed with the β-secretase 1 (BACE1) knockout (KO) mice [21] to generate homozygous PS2APP/BACE1^WT/WT^ or homozygous PS2APP/BACE1^KO/KO^ mice. Mice were housed with a 14-h light/10-h dark light cycle with ad libitum access to water and food. All animal experiments were approved by Genentech’s Institutional Animal Care and Use Committee and comply with the Institute for Laboratory Animals’ guidelines for the humane care and use of laboratory animals.

### In vivo dosing studies

Transgenic PS2APP or nontransgenic (Ntg) littermates were randomized into treatment groups and received a single intravenous (i.v.) dose of either crenezumab hIgG4 (20, 80, or 200 mg/kg) [11, 17, 22] or control hIgG4 (anti-glycoprotein D (gD), 40 mg/kg or 100 mg/kg) diluted in platform buffer (20 mM histidine, 240 mM sucrose, pH 5.5, 0.02% Tween 20) and were injected at a volume of 5 ml/kg. Five to 7 days after dosing, the animals were sacrificed and terminal plasma was collected via cardiac puncture prior to perfusion with phosphate-buffered saline (PBS); the right hemibrain was removed and drop-fixed in 4% paraformaldehyde. From the left hemibrain, the hippocampus, cortex, and cerebellum were dissected, weighed, and stored at − 80 °C. PS2APP-GFP-M mice were injected with a single intraperitoneal (i.p.) injection of crenezumab (120 mg/kg), and terminal plasma and brains were collected 48 h postdose. To determine the specificity of crenezumab’s binding to oligomeric Aβ, 6- to 7-month-old PS2APP/BACE1^WT/WT^ or PS2APP/BACE1^KO/KO^ mice were administered a single i.v. dose of crenezumab (80 mg/kg), and terminal plasma and brains were collected 7 days postdose. Mice dosed with anti-moAβ [23] received daily injections (100 mg/kg, i.p.) for 5 consecutive days, and plasma and brain tissues were collected 6 h after the final dose. Crenezumab-treated mice received a single injection of crenezumab (80 mg/kg, i.v.) and were sacrificed 5 days postdose.

### Immunohistochemistry

The right hemibrain was drop-fixed in 4% paraformaldehyde for 48 h and then transferred to 30% sucrose in PBS. Free-floating sagittal cryosections (35 μm) of the mouse brain were washed in PBS and then PBS-Triton X100 (PBST, 0.1%) and then blocked in PBST (0.3%) with 5% bovine serum albumin (BSA) and incubated overnight with primary antibodies diluted in 1% BSA in PBST (0.3%) at 4 °C. Goat anti-human IgG-Alexa594 (or Alexa555, 1:100–1:500; Thermo-Fisher, Waltham, MA) was used to localize the administered human antibody. Plaques were detected using the Aβ fluorescent marker methoxy-X04, and oligomeric Aβ was detected with mouse anti-human Aβ (mAβ-M 1:2000; Agrisera, Sweden) antibody. BACE1 was detected with rabbit anti-BACE1 (D10E5, 1:1000; Cell Signaling, Danvers, MA, USA) antibody, microglia with rabbit anti-Iba1 (1:1000; Wako, Richmond, VA, USA), and dystrophic neurites with rabbit anti-lysosomal-associated membrane protein 1 (LAMP1; 1:1000; abcam, San Francisco, CA, USA).

### Immunoprecipitation and Western blotting

Immunoprecipitations were carried out using synthetic preparations of Aβ 1–42 peptide (Aβ_42_), which was pre-formed into Aβ oligomers, or soluble brain homogenates from PS2APP which contain endogenous Aβ. Aβ oligomers were made by resuspending 0.5 mg of HFIP–treated Aβ_42_ (rPeptide, A-1163-1) in 450 μl of 150-mM ammonium hydroxide (pH 10.5) and incubating overnight at 4 °C. pH was neutralized by adding 50 μl of 3 M Tris (pH 7.2). Brains from nontreated 13- to 16-month-old male PS2APP mice or hippocampi from crenezumab-treated PS2APP/BACE1^WT/WT^ and PS2APP/BACE1^KO/KO^ mice were isolated and homogenized in 10 volumes of Tris-buffered saline (TBS) with Roche Phosphatase and Complete, Ethylenediaminetetraacetic acid (EDTA)-free, protease inhibitor cocktail tablets using a Qiagen TissueLyser II (2 × 3 min at 30 Hz). Samples were then centrifuged at 20,000×*g* for 20 min. Supernatant was collected for immunoprecipitation. The extracts were precleared with 50 μl of protein G Dynabeads (Invitrogen, Waltham, MA, USA) overnight at 4 °C. Five micrograms of antibody-bead complexes (gD, 6E10, 4G8, crenezumab, or moAβ) was incubated with the cleared supernatant overnight at 4 °C.

The beads were washed three times with TBS. Immunoprecipitated samples run on native PAGE were eluted in 150 mM ammonium hydroxide (pH 10.5) and neutralized with 3 M Tris to pH 7.2, a procedure previously described to result in high recovery of Aβ complexes without changing the size forms of the eluted species [24]. Samples were run with 1% digitonin on 4–16% Novex Bis-Tris gels (Invitrogen) at 150 V for 115 min. The first 30 min of the run contained dark blue cathode buffer (0.02% G-250) and then replaced with light blue cathode buffer (0.002% G-250) for the remainder of the run. Gels were soaked in 0.5% SDS for 30 min at 37 °C and transferred to polyvinylidene difluoride membranes (iBlot; Invitrogen). Blots were soaked in 8% acetic acid for 15 min to fix the proteins and then soaked in methanol briefly to destain. Membranes were boiled for 5 min to increase Aβ antigen exposure, blocked in 5% milk in TBS plus Tween 20, and probed with 6E10 (human Aβ 3-8, SIG-39320, Covance, Richmond, VA, USA) and 4G8 (Aβ 17-24, SIG-39220, Covance) antibodies for detection of amyloid precursor protein (APP) and Aβ, an antibody specific for the carboxy-terminal 20 residues of APP for detection of α- or β-carboxy-terminal fragments (SIG-39152, Covance) or an amino-terminal-specific antibody for APP (22C11, a.a 66-81, Millipore, Bedford, MA, USA) by chemiluminescence (BioRad Gel Doc).

### Fluorescent microscopy

Whole slide images are captured at × 20 using a Pannoramic 250 (3D Histech, Hungary) equipped with a PCO.edge camera (Kelheim, Germany), Lumencor Spectra X (Beaverton, OR), and Semrock filters (Rochester, NY) optimized for 4′6-diamidino-2-phenylindole, dihydrochloride (DAPI), tetramethylrhodamine isothiocyanate (TRITC), and cyanine 5 (Cy5) fluorophores. Ideal exposure for each channel is determined based on samples with the brightest intensity and is set for the whole set of slides to run as a batch. Images were also captured at × 20 using a Leica DM5500B light microscope using Leica Application Suite Advanced Florescence software (LAS AF4.0). Confocal images were taken using a × 20 or × 40 oil objective on a Zeiss LSM800 confocal laser scanning microscope using the Zen2.3 software. Quantification of mossy fiber staining was performed by measuring integrated density from two to four sections per animal using ImageJ (NIH).

### In vivo antibody pharmacokinetics (PK) and Aβ enzyme-linked immunosorbent assay (ELISA) measurements

#### Tissue preparation

Cerebellum samples were weighed and homogenized in 300 μl of 1% NP-40 (with Roche complete ETDA-free protease inhibitor cocktail) using a Qiagen TissueLyser II (2 × 3 min at 30 Hz). Samples were then placed on ice for 20 min and then centrifuged at 20,000×*g* for 20 min. Supernatant was collected and stored at − 80 °C until used for PK assay.

#### Pharmacokinetics assays

Antibody concentrations in mouse plasma and brain samples were measured using ELISA. NUNC 384-well Maxisorp immunoplates (Neptune, NJ, USA) were coated with F (ab′)2 fragment of sheep anti-human IgG, Fc fragment-specific polyclonal antibody (Jackson ImmunoResearch, West Grove, PA, USA) overnight at 4 °C. Plates were then blocked with PBS containing 0.5% BSA for 1 h at room temperature. Each antibody (control IgG and anti-moAβ and crenezumab) was used as a standard to quantify the respective antibody concentrations. After the plates were washed with PBS containing 0.05% Tween 20 using a microplate washer (Bio-Tek Instruments, Inc., Winooski, VT), standards and samples diluted in PBS containing 0.5% BSA, 0.35 M sodium chloride (NaCl), 0.25% 3-[(3-Cholamidopropyl)dimethylammonio]-1-propanesulfonate hydrate (CHAPS), 5 mM EDTA, 0.05% Tween 20, and 15 ppm Proclin were incubated on plates for 2 h at room temperature with mild agitation. Bound antibody was detected with horseradish peroxidase-conjugated F (ab′)_2_ goat anti-human IgG, Fc fragment-specific polyclonal antibody (Jackson ImmunoResearch). Finally, plates were developed using the substrate 3,3′,5,5′-tetramethyl benzidine (KPL, Inc., Gaithersburg, MD, USA). Absorbance was measured at a wavelength of 450 nm with a reference of 630 nm on a Multiskan Ascent reader (Thermo Scientific, Hudson, NH, USA). Concentrations were determined from the standard curve using a four-parameter nonlinear regression program. The assay had lower limit of quantitation values of 13.7 ng/ml in the plasma and 1.37 ng/ml in the brain.

#### Pharmacodynamics assays

Aβx-40 and Aβx-42 concentrations in mouse plasma samples were measured using an ELISA similar to that used for the PK analysis described above. Briefly, rabbit polyclonal antibody specific for the C terminus of Aβ_40_ or Aβ_42_ (Millipore) was coated onto plates, and biotinylated monoclonal anti-Aβ1-16 (6E10; Covance, Dedham, MA) was used for detection. The assay had lower limit of quantification values of 15.6–23.4 pg/ml in plasma.

### Statistical analysis

One-way analysis of variance (ANOVA) followed by Tukey’s multiple comparison tests or Student’s unpaired *t* test (two-sided) was performed using either Prism 6.0 (GraphPad Software, San Diego, CA, USA) or JMP12.2.0 (SAS Institute Inc., Cary, NC, USA) software.

## Results

### Crenezumab immunoprecipitates oligomeric Aβ in vitro

**Fig. 1:**
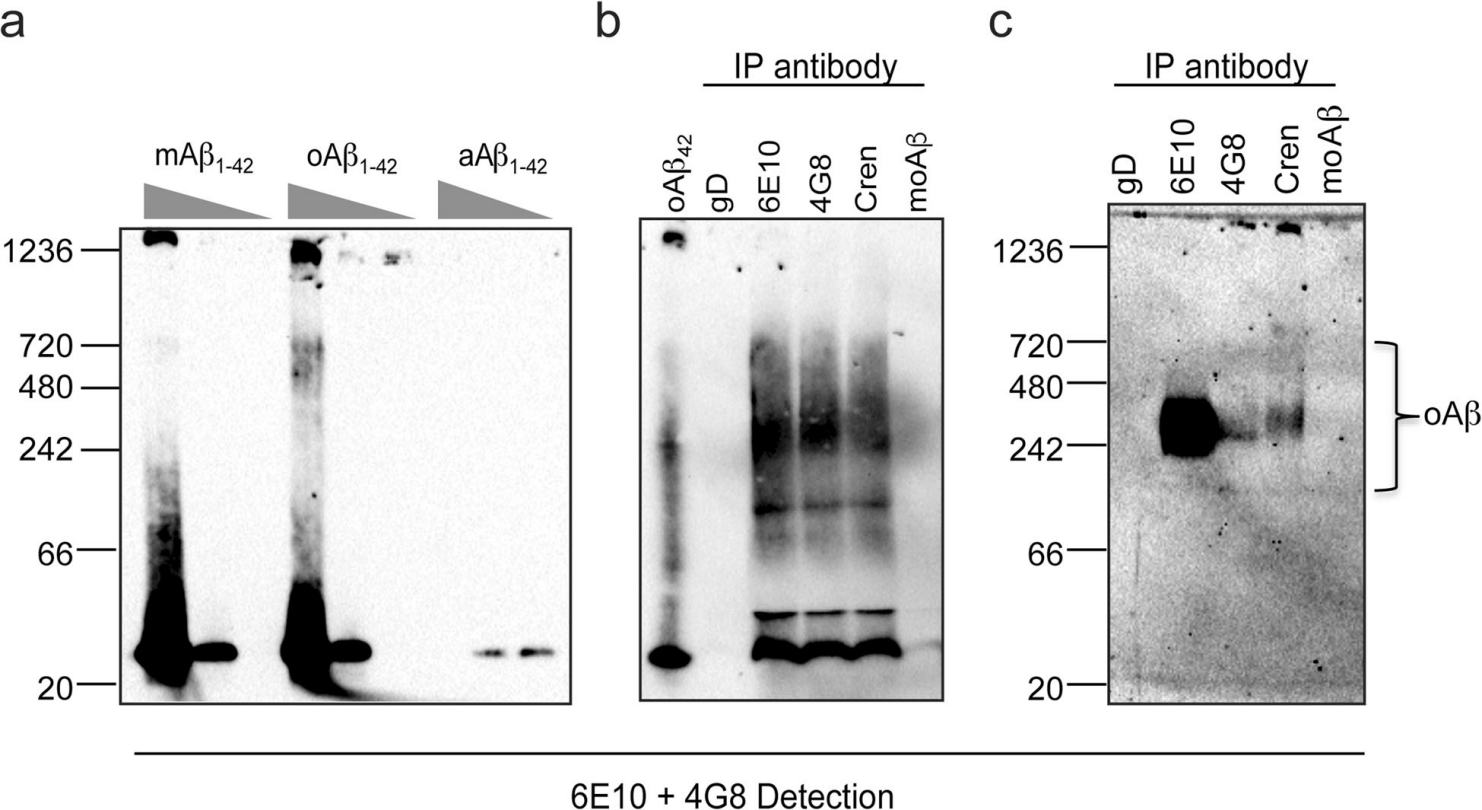
Crenezumab recognizes Aβ oligomers from in vitro and in vivo sources. Pre-formed (m)onomericAβ_42_, (o)ligomericAβ_42_, or (a)ggregatedAβ_42_ were run on native PAGE at 1000, 500, and 250 ng per lane to visualize Aβ_42_ banding patterns (**a**). Note that aAβ was too large to enter the gel. Antibodies were incubated with pre-formed Aβ_42_ oligomers overnight at 4 °C. To visualize nondenatured oligomers, immunoprecipitated (IP) eluates using were run on native PAGE. Crenezumab recognizes both low molecular weight oligomers between 20 and 50 kDa and high molecular weight (HMW) oligomers between 250 and 700 kDa (**b**). Anti-Aβ IPs from the soluble fraction of PS2APP mouse brain homogenates were run on native PAGE. Crenezumab recognizes HMW oligomers (**c**). 6E10 and 4G8 were used as detection antibodies on all blots

To investigate crenezumab’s binding to Aβ oligomers, we performed immunoprecipitation experiments either with synthetic pre-oligomerized Aβ_42_ preparations or with endogenous Aβ from the soluble fraction of plaque-enriched PS2APP brain homogenates. Samples were separated on native nondenaturing gels, which allow aggregated oligomers to remain relatively intact. We observed that pre-formed Aβ_42_ oligomers, but not monomers, could be specifically resolved as a high molecular weight smear on these gels (Fig. 1a). Immunoprecipitations of pre-formed synthetic Aβ_42_ by crenezumab, as well as other well-characterized commercial pan anti-Aβ antibodies (6E10 and 4G8), efficiently pulled down large oligomeric forms of Aβ (~ 66–720 kDa, Fig. 1b). Lower molecular weight species that may represent a mixture of residual monomers or low-*n* oligomeric forms (dimers, trimers) were also detected in the pull down. In comparison, a monomeric Aβ-preferring antibody (anti-moAβ) [23] that has an ≈ 25-fold higher affinity to monomeric Aβ than crenezumab and nondetectable binding to oligomers (Table 1) failed to pull down much oligomeric Aβ (Fig. 1b). Isotype control antibody (anti-gD) did not engage Aβ.

**Table 1:** In vivo antibody PK and in vitro biacore binding properties

Molecule	Dose (i.v.)mg/kg	Isotype	*C*_max_(μg/ml)	AUC_last_(μg/ml day)	CL_obs_(ml/day/kg)	Biacore monomericAβ Kd (nM)	Biacore oligomericAβ Kd (nM)
Crenezumab	20	hIgG4	296	882	19.6	5	0.5
Anti-moAβ [23]	20	hIgG1	319	113	176.4	0.2	Not detected
Anti-gD	20	hIgG1	410	1457	7.6	–	–

*PK* pharmacokinetics, *i.v.* intravenous, *C*_*max*_ maximum concentration, *AUC*_last_ area under the curve from the time of last dosing to the last measurable concentration, *CL*_obs_ total body clearance, *Aβ* amyloid β, *hIgG* humanized immunoglobulin G, *moAβ* monomeric amyloid β, *gD* glycoprotein D

Crenezumab was also used to immunoprecipitate endogenous Aβ from TBS-soluble PS2APP brain homogenates, an amyloidosis mouse model of AD that expresses mutant forms of hAPP (K670N/M671L) and presenilin-2 (PS2N141I). Samples were separated on nondenaturing gels and again showed that crenezumab, unlike moAβ or isotype control antibodies, immunoprecipitated oligomeric forms of Aβ that resolved as high molecular weight oligomers (Fig. 1c). These results suggest that crenezumab is able to bind to high molecular weight Aβ oligomers generated in vivo. Pan anti-Aβ antibodies 6E10 and 4G8 also immunoprecipitated high molecular weight Aβ oligomers to different degrees, possibly due to recognition of distinct epitopes, which may be differentially exposed in oligomers, as well as due to differential affinities for various Aβ species. Size-exclusion chromatography of TBS-soluble brain extracts confirmed that the majority of Aβ species found in these brain samples was of a high molecular weight, consistent with being oligomeric in nature (i.e., eluted in or near the void volume of column, data not shown). Together, these results suggest that crenezumab is able to bind and engage a range of oligomeric Aβ forms, including endogenous oligomers thought to mediate neurotoxicity in AD [2–4, 8, 25–29].

### In vivo immunolocalization of crenezumab to regions surrounding the dense plaque core in PS2APP transgenic mice

**Fig. 2:**
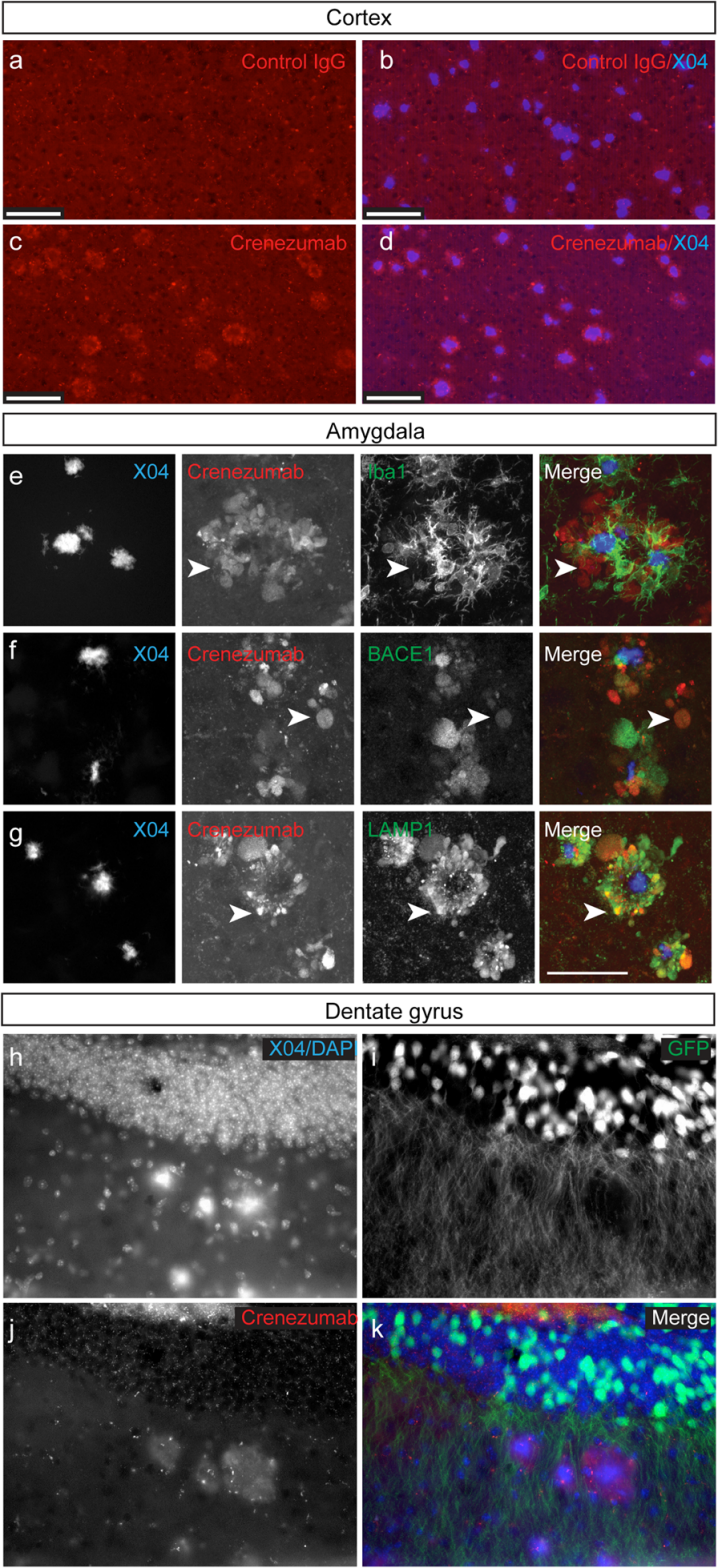
In vivo-dosed crenezumab binds in a halo around amyloid plaques and to dystrophic neurites in PS2APP mice. In vivo-dosed crenezumab (200 mg/kg, i.v.) was visualized 7 days postdose with anti-hIgG-Alexa594 antibody (red), and plaques were stained with methoxy-X04 (blue). Representative epifluorescent images of plaque-associated halo of staining by crenezumab alone (**c**) and with plaques (**d**) in the cortex. Note the absence of staining in the control-injected (control IgG, gD) mice around plaques (**a**, **b**). In the amygdala (**e**–**g**), confocal z-stacked images show crenezumab binding was prominent around the core of the plaque but in regions not covered by microglia (**e**) (labeled with Iba1, green). This staining pattern was reminiscent of dystrophic neurites and was confirmed by co-staining of crenezumab (80 mg/kg, i.v., red) with markers of dystrophic neurites including BACE1 (green, **f**) and LAMP1 (green, **g**). Arrowheads indicate example regions of overlap. In vivo-dosed crenezumab (**j**, **k**, red, 120 mg/kg, IP) was localized to regions between methoxy-X04-labeled plaques (**h**, **k,** blue) and GFP-labeled dendrites (**i**, **k**, green) in the dentate gyrus of PS2APP-GFP (line M) mice (2 days postdose). Scale bar, 200 μm (**a**–**d**) and 50 μm (**e**–**g**)

To characterize the in vivo binding properties of crenezumab, we dosed PS2APP mice with a single i.v. dose of crenezumab hIgG4 (20, 80, or 200 mg/kg) or control hIgG4 (anti-gD, 100 mg/kg) and collected brain tissue 7 days postdose for immunohistochemical analysis. Doses were selected to yield comparable exposure, on the basis of preliminary pharmacokinetic studies suggesting that systemic clearance of crenezumab in this mouse model is approximately twofold faster than that of the control antibody (Table 1). In vivo binding of crenezumab was assessed by immunostaining brain sections for hIgG, and plaques were identified by Methoxy-X04 staining. In the cortex, crenezumab localized to the periphery of amyloid plaques, and little to no binding to the dense core of the plaque was observed; control IgG4 binding was minimal and showed little to no specificity in plaque-containing regions (Fig. 2a–d). The halo staining of crenezumab to plaques was prominent throughout the brain but highest in regions that started to deposit plaques earliest (i.e., the subiculum and amygdala). Immunostaining of crenezumab around plaques in the amygdala, for example, was robust and tended to concentrate to regions of the plaque not surrounded by Iba1+ microglia (Fig. 2e), suggesting that oligomeric Aβ, being detected by crenezumab, may accumulate in regions near the plaque where microglia are absent, in a so-called hot-spot [30].

Soluble oligomeric forms of Aβ are considered more neurotoxic than dense core plaques [1–3, 5, 7, 8, 31, 32] and seem to play a greater role in Aβ-induced neurotoxicity, including synaptic spine loss and the formation of neuritic dystrophies [8, 26, 33, 34], which are large swellings of axons/neurites that occur in close proximity to amyloid plaques [34, 35]. Numerous proteins have been found to accumulate in dystrophic neurites, including LAMP1, ubiquitin, synaptophysin, APP, and BACE1 [30, 36, 37]. Accumulation of BACE1 in dystrophic neurites is thought to contribute to increased Aβ production locally near plaques [36]. To determine whether crenezumab binding to the halo region around plaques associates with markers of neuritic dystrophies, we performed confocal imaging of co-stained sections from in vivo-dosed animals for crenezumab and BACE1 (Fig. 2f) or LAMP1 (Fig. 2g) and found the crenezumab signal was localized proximal to both BACE1 and LAMP1, indicating a close spatial relationship between the Aβ forms that are bound by crenezumab surrounding the plaque core. It is unlikely that the in vivo-dosed crenezumab is able to enter the intracellular space of the dystrophic neurite; rather, crenezumab appears to be decorating the outside of these structures marked by BACE1 or LAMP1. Moreover, in PS2APP mice neuronally expressing GFP to visualize dendritic processes, crenezumab binding was found to concentrate within regions surrounding methoxy-X04–stained plaques that were devoid of dendritic processes (Fig. 2h–k), reflecting Aβ-induced neurotoxicity.

Together, these results suggest that crenezumab is selectively binding, in vivo, to peripheral regions around the plaque core where there is believed to be a high concentration of soluble oligomeric Aβ that contributes to neuronal and synaptic dysfunction.

### In vivo-dosed crenezumab does not bind to vascular amyloid

**Fig. 3:**
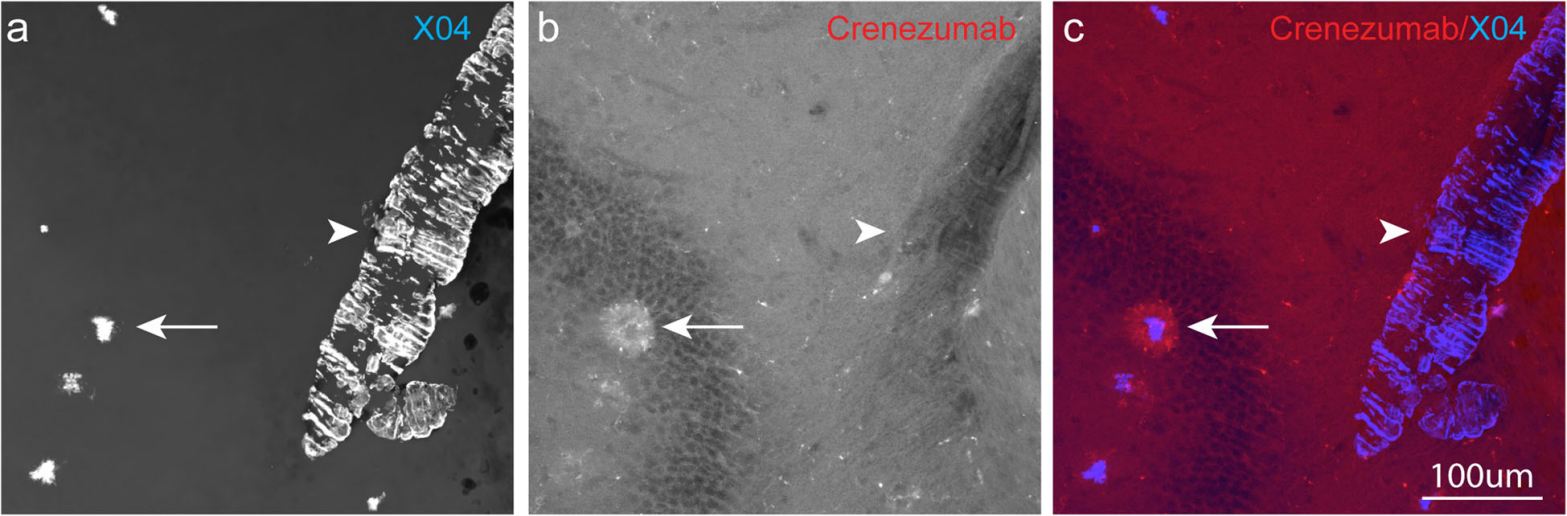
In vivo-dosed crenezumab does not bind to vascular amyloid in PS2APP mice. Representative confocal × 40 images (z-stack maximum projection) of parenchymal amyloid plaques (arrow) and vascular amyloid (arrowhead) stained with methoxy-X04 (**a**, **c**, blue). Note the selective staining of in vivo-dosed crenezumab (200 mg/kg, i.v.) (**b**, **c**, red) to the peri-plaque region and the absence from the vascular amyloid. Scale bar, 100 μm

Similar to parenchymal plaques, amyloid deposits in the brain vasculature are often observed in patients with AD. These vascular deposits are recapitulated in PS2APP mice and were readily detectable by methoxy-X04 staining. However, we found no evidence of vascular amyloid binding by crenezumab even when PS2APP mice were dosed up to 200 mg/kg (Fig. 3a–c). The lack of crenezumab binding to the cerebral amyloid angiopathy is consistent with its lack of binding to the parenchymal plaque core, both structures that are detected by methoxy-X04 staining. This suggests that crenezumab does not interact with this form of highly aggregated fibrillar Aβ. These findings may have important relevance to the reported low incidence of ARIA-E in crenezumab-treated patients [11, 17, 22].

### In vivo-dosed crenezumab binds to mossy fiber axons in the hippocampus

**Fig. 4:**
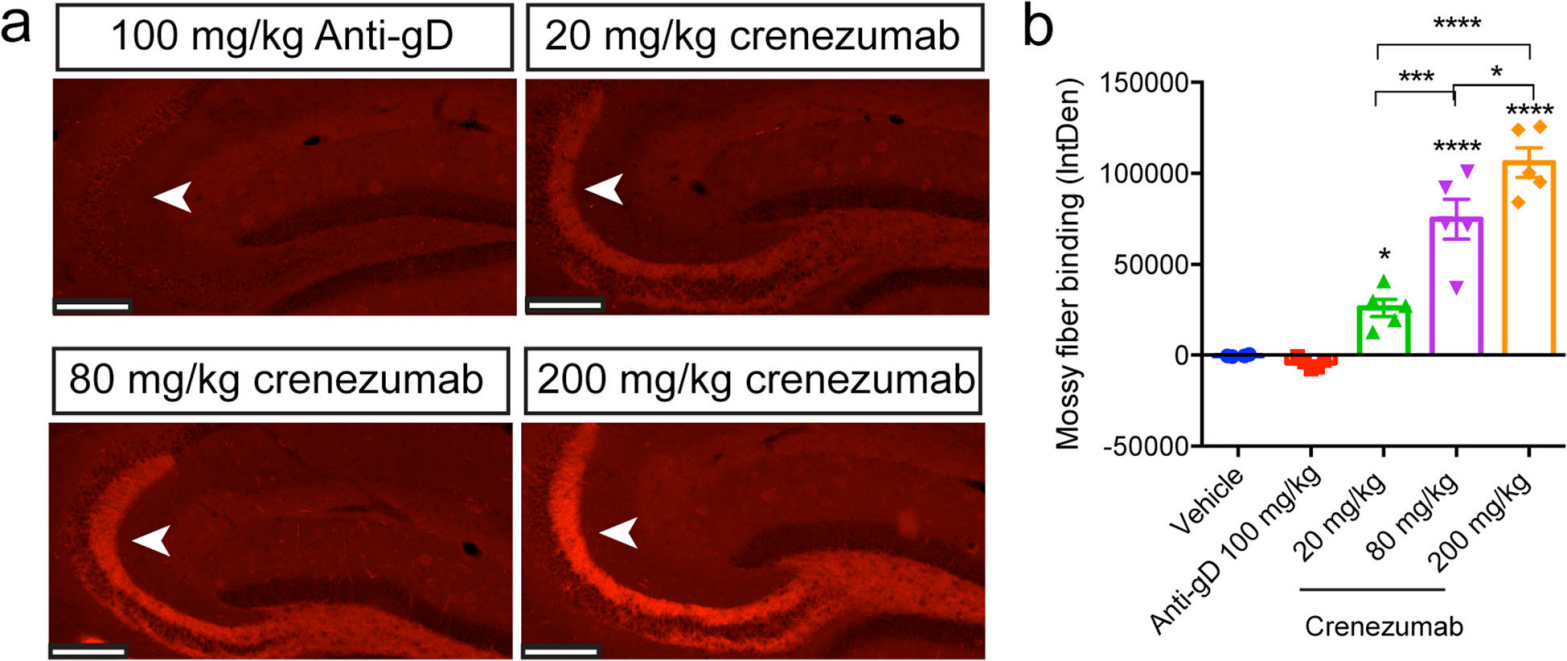
In vivo-dosed crenezumab binds to the mossy fibers in PS2APP mice. In vivo-dosed crenezumab, but not control IgG (anti-gD IgG4), dose-dependently binds to the mossy fiber axons in the hippocampus of PS2APP mice. Representative epifluorescent images of mossy fiber binding by crenezumab in PS2APP mice (**a**). Quantification of mossy fiber binding integrated density (IntDen) found a significant treatment effect (**b**) (ANOVA: *F*_4,19_ = 50.10, *p* < 0.0001). ANOVA followed by Tukey’s multiple comparison test: *p < 0.05, ***p < 0.001, ****p < 0.0001 as indicated or to control IgG (anti-gD)

In vivo dosing of crenezumab led to the novel finding that prominent immunoreactivity was observed in the mossy fiber tract of the hippocampus in PS2APP mice (Fig. 4a, b). The mossy fibers are the axons of the dentate granule cells that terminate in the hilus and in the stratum lucidum of the CA3 region [38–40]. Mossy fiber binding was specific to crenezumab, as no staining was observed in the control IgG-injected animals (Fig. 4a). Crenezumab binding to the mossy fibers was dose dependent and significantly greater than that in vehicle and control IgG-injected animals (Fig. 4a, b). The lack of methoxy-X04 staining in the mossy fibers suggests that crenezumab’s binding was not to fibrillar Aβ, but likely to soluble Aβ species.

**Fig. 5:**
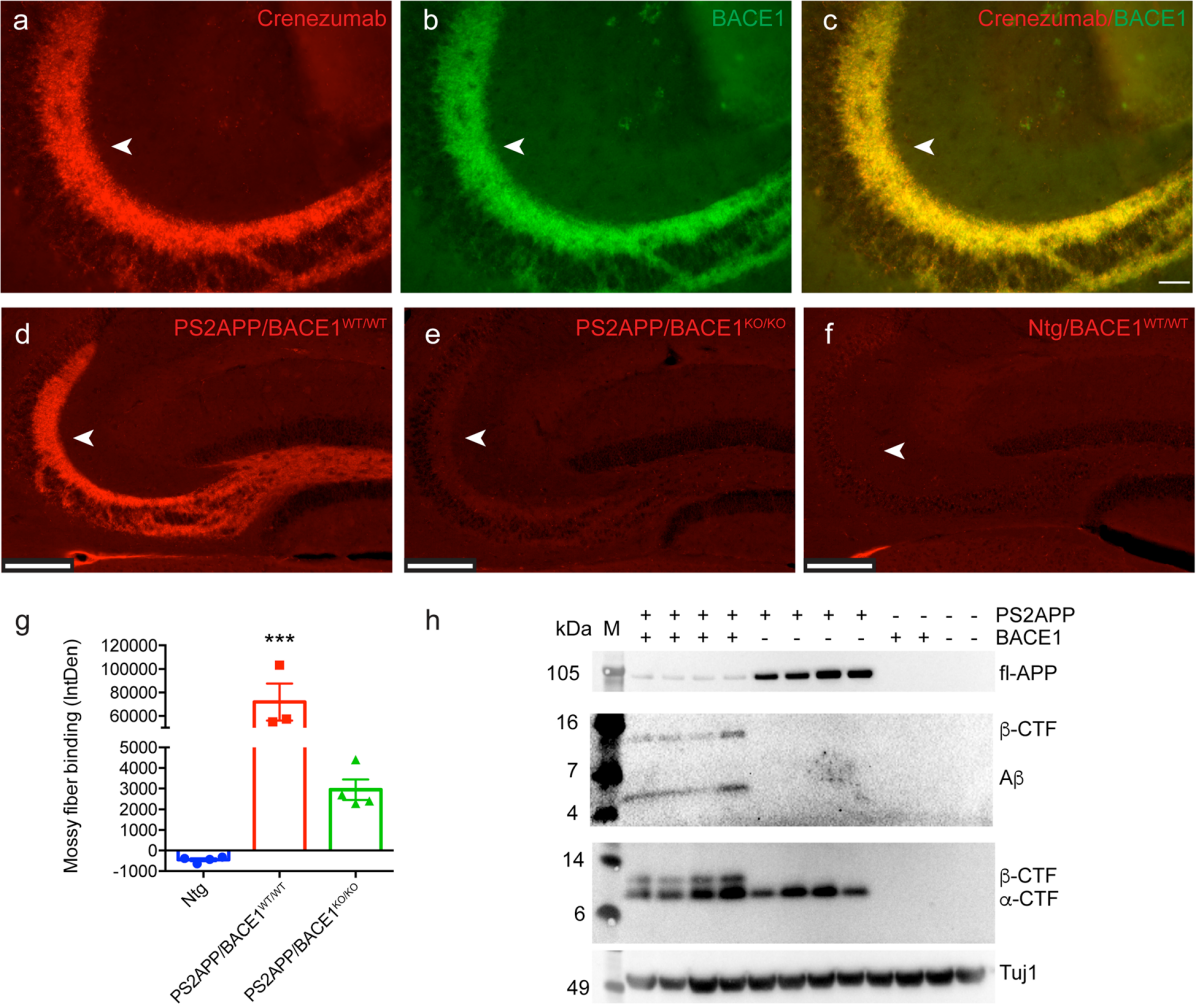
Crenezumab binding to the hippocampal mossy fibers is Aβ dependent. Representative epifluorescent images of in vivo-dosed crenezumab (80 mg/kg) binding to the mossy fibers (**a**) of PS2APP mice (arrows). Immunostaining for BACE1 shows strong binding in the mossy fibers (**b**) that overlap with crenezumab staining (**c**, merge). Scale bar = 50 μm. In vivo-dosed crenezumab (80 mg/kg) staining to the mossy fibers in the PS2APP/BACE1^WT/WT^ mice (**d**) was nearly completely absent in PS2APP/BACE1^KO/KO^ (**e**) compared with Ntg/BACE1^WT/WT^ (**f**) mice. Scale bar, 200 μm. **g** Significant differences in mossy fiber binding were found between the groups (ANOVA: *F*_2,8_ = 29.16, *p* < 0.001) *n* = 3–4/group. ANOVA followed by Tukey’s multiple comparison test. ***p < 0.001 versus all others. **h** Western blots of full-length/soluble APP and Aβ (detected by 4G8 and 6E10) and α/β–C-terminal fragment (detected by SIG-39152) from soluble hippocampal TBS homogenates from PS2APP/BACE1^WT/WT^, PS2APP/BACE1^KO/KO^, and Ntg/BACE1^WT/WT^ mice. β-Tubulin (Tuj1) was used as a loading control. M, molecular weight marker

Neuronal BACE1 initiates the processing of APP and is hypothesized to facilitate Aβ overproduction in AD-affected brains [36, 37]. BACE1 is expressed in neurons throughout the brain and is highly expressed in the mossy fiber axons of the hippocampus [36, 37, 41], suggesting that a high level of APP processing could lead to Aβ accumulation, driving oligomer generation in this region of the hippocampus. To confirm that the crenezumab binding to the mossy fibers was dependent on Aβ and not binding to APP or soluble APP fragments, we crossed the PS2APP mice with BACE1 KO mice. PS2APP/BACE1^WT/WT^ and PS2APP/BACE1^KO/KO^ mice were dosed with crenezumab (80 mg/kg), and brain samples were collected 7 days postdose. First, we observed that in vivo-dosed crenezumab localized strongly to mossy fiber tracts with positive immunostaining for BACE1, suggesting that crenezumab is immunodecorating the mossy fiber axons of PS2APP/BACE1^WT/WT^ mice (Fig. 5a–c). Interestingly, crenezumab binding often appeared as bright puncta in the CA3 region; we speculated that crenezumab may concentrate around the large mossy fiber presynaptic terminals [38, 41]. Next, when compared with PS2APP/BACE1^WT/WT^ mice (Fig. 5d), crenezumab binding to the mossy fibers was markedly reduced in PS2APP/BACE1^KO/KO^ mice (Fig. 5e), indicating that binding is dependent on BACE1 activity, thus indeed predominantly Aβ dependent. Quantification of the mossy fiber binding relative to surrounding neuropil (Fig. 5g) did show, however, that low levels of crenezumab binding remained in the mossy fibers of the PS2APP/BACE1^KO/KO^ mice, which could represent binding to APP or to a low level of Aβ produced by alternative proteases, since this signal was completely absent in the Ntg mice (Fig. 5f). We also confirmed by Western blot that the BACE1 deletion blocked any detectable APP processing and Aβ production in the PS2APP mice. We found that BACE1 deletion caused an elevation in full-length/soluble APP and a complete loss of BACE1 cleavage products, including β–C-terminal fragment and Aβ (Fig. 5h).

**Fig. 6:**
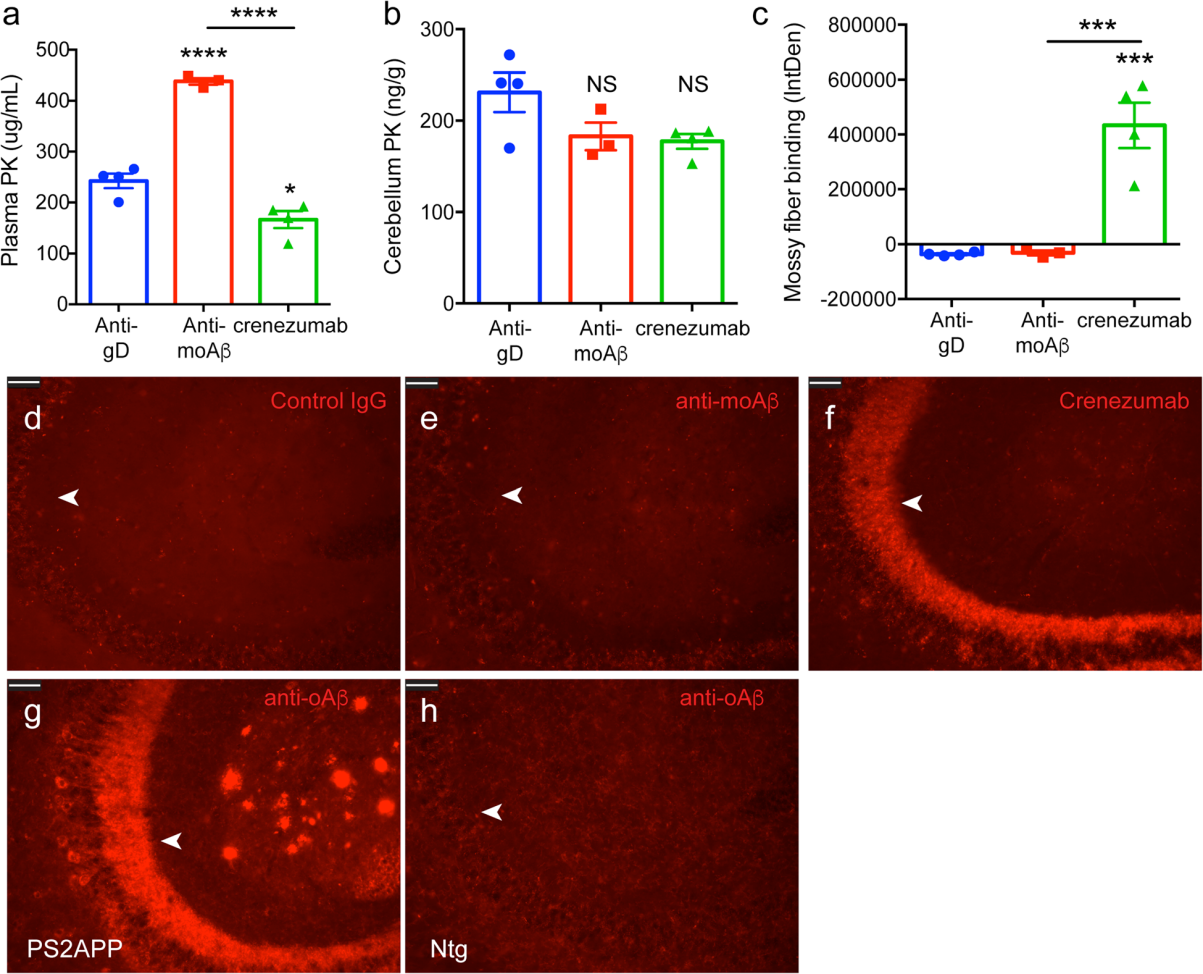
In vivo-dosed crenezumab binds to (o)ligomeric Aβ, not to (mo)nomeric Aβ, in the hippocampal mossy fiber tract. Plasma and cerebellum PK levels 6 h after the final day of dosing (100 mg/kg daily for 5 d) with an anti-moAβ (*n* = 3) or 5 days after a single injection of control IgG (anti-gD 40 mg/kg, *n* = 4) or crenezumab (80 mg/kg, *n* = 4) in PS2APP mice. ANOVA found a significant difference in plasma PK levels (**a**) (*F*_2,8_ = 86.90, *p* < 0.0001) but not in the cerebellum (**b**; not significant [NS]). Quantification (**c**) and representative epifluorescent images (**d**–**f**) of mossy fiber binding by crenezumab but not by control IgG or moAβ antibodies. ANOVA found a significant difference in binding (*F*_2,8_ = 26.84, *p* < 0.001). Representative images of ex vivo oAβ staining in the mossy fibers (arrows) of PS2APP mice (**g**), but not in Ntg mice (**h**), using an anti-oAβ antibody (mab-M). Scale bar, 50 μm. ANOVA followed by Tukey’s multiple comparison test: *p < 0.05, ***p < 0.001, ****p < 0.0001 as indicated or to control IgG

We next wanted to determine whether the mossy fiber binding represented soluble monomeric Aβ or oligomeric Aβ binding by crenezumab. To investigate this further, we dosed PS2APP mice with either crenezumab or a monomer-preferring anti-moAβ antibody. Preliminary studies (Table 1) found that the anti-moAβ antibody had fast clearance (176.4 ml/day/kg) compared with crenezumab (19.6 ml/day/kg) following a single dose in PS2APP mice. Therefore, to achieve comparable exposure levels in the brain, we needed to dose the PS2APP mice daily with the anti-moAβ (100 mg/kg) for 5 consecutive days and collect the brain and plasma samples 6 h after the final dose. Crenezumab (80 mg/kg) or an isotype control antibody (40 mg/kg) was given once, and tissues were collected 5 days postdose. At the end of the study, peripheral antibody concentrations of anti-moAβ were approximately twofold higher than crenezumab (Fig. 6a), due to the more frequent dosing. In the brain, both crenezumab and anti-moAβ had similar exposures, as desired (Fig. 6b); however, only crenezumab showed the characteristic mossy fiber binding unlike anti-moAβ (Fig. 6c–f). Together, these results suggest that in vivo-dosed crenezumab binds to oligomeric Aβ, not monomeric Aβ.

To further confirm that the mossy fibers contained oligomeric Aβ, we immunostained brain sections from PS2APP and Ntg mice ex vivo with an antibody preferential for oligomeric Aβ (mAb-M) and found prominent staining in the mossy fibers of only PS2APP mice (Fig. 6g) but not Ntg (Fig. 6h). This result is consistent with previous studies in another AD mouse model using a different oligomeric Aβ antibody [37]. Overall, these findings suggest that crenezumab is binding to soluble oligomeric Aβ, not to monomeric Aβ in the mossy fiber tract.

### Measures of peripheral target engagement correlate with crenezumab binding to the mossy fibers

**Fig. 7:**
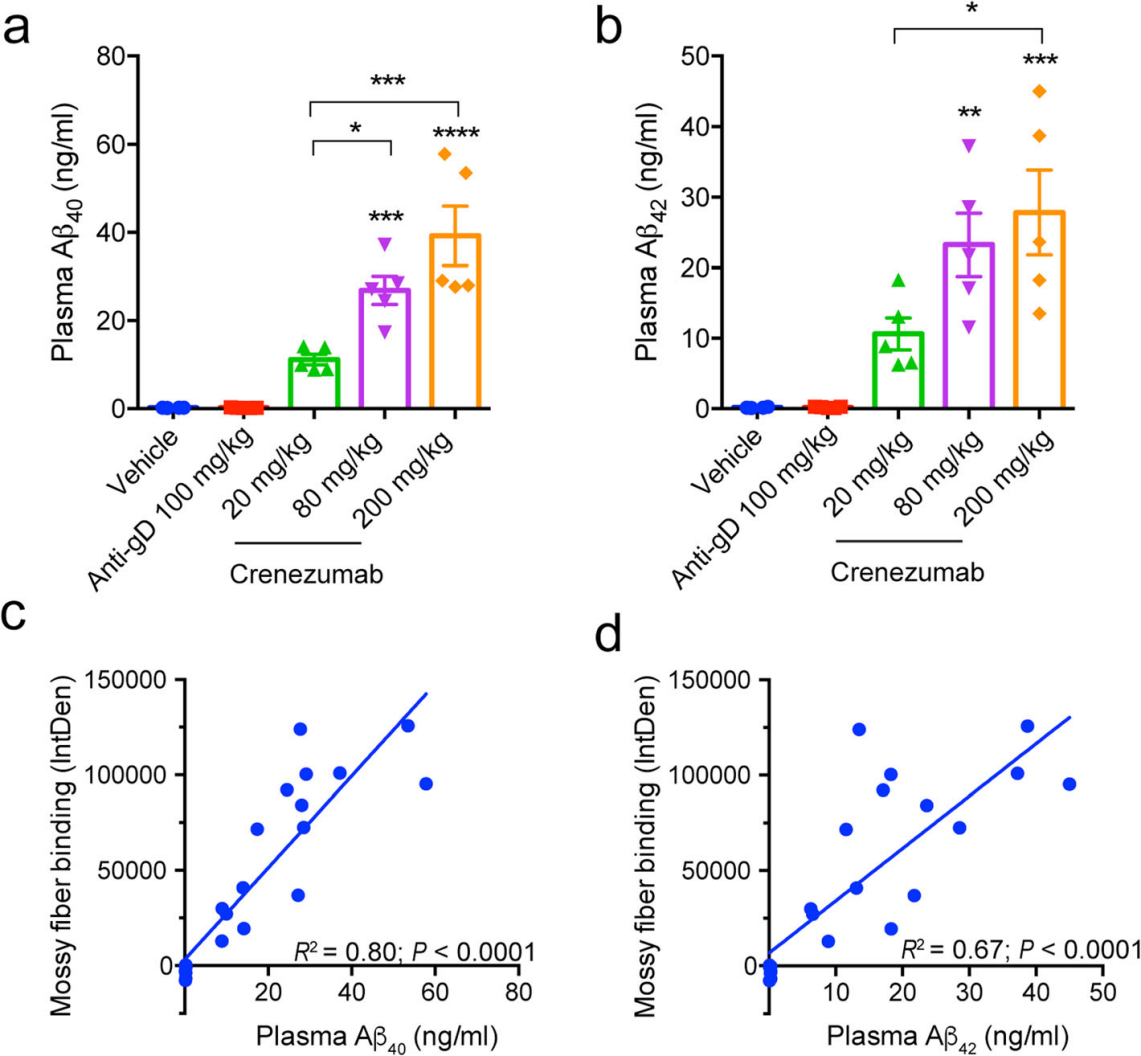
In vivo crenezumab-mediated target engagement correlates between the periphery and brain. Significant elevations in plasma Aβ_40_ (**a**) (ANOVA: *F*_4,19_ = 23.43, *p* < 0.0001) and Aβ_42_ (**b**) (ANOVA: *F*_4,19_ = 12.08, *p* < 0.0001) were found 7 days after crenezumab treatment. A significant correlation was observed between the elevations in plasma Aβ_40_ (**c**) (*R*^2^ = 0.08, *p* < 0.001) and Aβ_42_ (**d**) (*R*^2^ = 0.67, *p* < 0.001) and crenezumab mossy fiber binding (see Fig. 4) by linear regression. *N* = 5/group. ANOVA followed by Tukey’s multiple comparison test: *p < 0.05, **p < 0.01, ***p < 0.001, ****p < 0.0001 as indicated or to control IgG

To investigate crenezumab’s engagement of soluble Aβ species in the periphery, we measured changes in soluble Aβ in the plasma of crenezumab-treated PS2APP mice. We found a significant dose-dependent elevation in plasma Aβ_40_ (Fig. 7a) and Aβ_42_ (Fig. 7b) with crenezumab treatment compared with vehicle and control IgG-injected animals. This is consistent with reported elevations in plasma and cerebrospinal fluid (CSF) Aβ following crenezumab treatment in patients [11, 17, 22]. In the plasma, this likely represents stabilization of the Aβ/antibody complex, leading to reduced clearance rates of Aβ. However, it is not known if any relationship exists between such peripheral target engagement and central (brain) target engagement by crenezumab. To investigate this in our AD mouse model, we looked to see if there was any correlation between plasma Aβ and mossy fiber binding. Here, we found a significant positive correlation between plasma Aβ elevations and crenezumab mossy fiber binding for both Aβ_40_ (Fig. 7c; *R*^2^ = 0.80, *p* < 0.0001) and Aβ_42_ (Fig. 7d; *R*^2^ = 0.67, *p* < 0.0001), suggesting that measures of peripheral target engagement (plasma Aβ elevations) can correlate with in vivo brain target engagement (mossy fiber binding). These results also indicate that crenezumab binding to soluble Aβ in the plasma does not hinder the ability of antibody to bind to Aβ in the brain even with doses as high as 200 mg/kg in this mouse model.

## Discussion

The search for an effective treatment for AD has proven to be enormously challenging. Several therapeutic approaches targeting amyloid have been, or are being, tested in the clinic, including passive immunization with anti-Aβ antibodies [29], inhibition of Aβ production by blocking BACE1 activity [42–44], and modulation of gamma-secretase activity [44, 45]. As the disease etiology continues to be investigated, it has become clear that Aβ can exist in multiple forms and conformations (monomer, oligomer, and insoluble fibrils/plaque) and that anti-Aβ antibodies directed against these different forms of Aβ may have varying therapeutic benefit.

In this study, we aimed to further characterize the binding properties of our anti-Aβ IgG4 mAb crenezumab both in vitro and in vivo. In vitro binding studies with synthetic and brain-derived oligomeric preparations confirm crenezumab’s ability to bind and immunoprecipitate multiple forms of Aβ, including both low (dimers and trimers) and high (~ − 700 kDa) molecular weight oligomers in addition to monomers. In vivo dosing experiments using the PS2APP mouse, a transgenic mouse model of AD, demonstrated that crenezumab binding localized to brain areas with putative high concentrations of Aβ oligomers, the form of Aβ thought to be the most neurotoxic [2–4, 25]. These areas included the periphery of amyloid plaques and hippocampal mossy fibers. Crenezumab’s staining pattern is markedly different from plaque-preferring anti-Aβ antibodies, such as aducanumab and gantenerumab, that have been reported to show strong immunostaining within the dense core, not the periphery, of the amyloid plaque but no mention of or reported binding to the mossy fibers [16, 46]. This could be due to potential differences in each anti-Aβ antibody’s in vivo Aβ binding profile and mechanism of action; however, we cannot rule out differences in the types of Aβ species generated in by different transgenic hAPP mouse models used.

Crenezumab binding to the halo surrounding plaques is consistent with findings that there is a synaptotoxic halo in close proximity surrounding plaque cores (within 50 μm) that consists of Aβ oligomers [8, 9, 47, 48]. Indeed, PS2APP mice have significantly reduced synaptic spine densities within this halo [19, 49], and we now have shown that crenezumab selectively binds within regions of dendritic loss surrounding the plaque core. Crenezumab was also found to bind to regions surrounding amyloid plaques that are not covered by Iba1+ microglia. These regions are reported to be “hot-spots” that actively incorporate soluble Aβ and contribute to Aβ-induced neurotoxicity and axonal dystrophy [30]. As noted earlier, soluble oligomeric forms of Aβ are associated with greater neurotoxicity compared with dense core plaques [2–4, 25]. Crenezumab binding was also found to associate closely with regions of neuritic dystrophy (identified by staining for LAMP1 and BACE1). Both LAMP1 and BACE1 are known to accumulate in dystrophic neurites, perhaps indicating dysfunctional axonal transport and function [30, 36, 37]. The accumulation of BACE1 to dystrophic neurites is hypothesized to contribute to local APP processing and soluble Aβ production that “feed” the plaques and contribute to synaptic dysfunction [36, 37]. Crenezumab binding and immunodecorating to regions of dystrophic neurites suggests that this may also be a region of concentrated oligomeric Aβ accumulation. For instance, blocking the continued production of soluble Aβ in plaque-bearing transgenic hAPP mice using a tet-off system reduced soluble levels of oligomeric Aβ, ameliorated synaptic loss near plaques, and reduced neuritic dystrophies in addition to improving cognitive function [50], all of which were further enhanced with anti-Aβ antibody treatment [51].

In vivo dosing studies also unveiled the novel mossy fiber binding by crenezumab. This binding was dose dependent and specific. Results showing a lack of methoxy-X04 and anti-moAβ antibody binding to this region, although binding by another oligomer-preferring antibody was observed, provide further evidence that crenezumab binding was likely to oligomeric Aβ. The presence of high BACE1 expression in the mossy fibers, again, suggests a region of concentrated oligomeric Aβ production. In addition, crenezumab staining was markedly reduced in PS2APP-BACE1^KO/KO^ mice, indicating that the mossy fiber staining was indeed an interaction with soluble extracellular Aβ and not with full-length or soluble APP.

Identifying biomarkers in the CSF or periphery that are translatable to in vivo target engagement in the brain for crenezumab, or any other anti-Aβ antibody, will be of critical importance for clinical trial investigations. We have found that elevations in plasma Aβ_40_ and Aβ_42_ levels following crenezumab treatment in PS2APP mice recapitulate elevations observed in crenezumab-treated patients [11, 17, 22], likely representing a change in clearance properties of Aβ once bound by crenezumab given the otherwise rapid clearance of Aβ alone [52, 53]. The elevation in PS2APP plasma Aβ levels significantly correlated with the intensity of crenezumab’s mossy fiber binding, suggesting that peripheral evidence of target engagement correlates with target engagement in the brain. We can also conclude that the peripheral target engagement by crenezumab did not act as a “sink,” thereby reducing the amount of free antibody able to enter the brain and bind Aβ.

One of the most commonly reported adverse events associated with anti-Aβ antibodies in clinical trials is ARIA-E [29, 54–56]. Crenezumab, to date, has been shown to have a low occurrence of ARIA in clinical trials [11, 17, 22] which may be partially attributed to its reduced effector function on an IgG4 backbone [11]. Anti-Aβ antibodies that reportedly bind to insoluble aggregated forms of Aβ have a higher incidence of ARIA in clinical trials [16, 57, 58] and, when injected into mouse models of AD, bind to vascular amyloid in addition to the fibrillar core of amyloid plaques [16, 59]. In this study, we identified that crenezumab does not bind to vascular amyloid or to the fibrillar dense core of plaques, suggesting that vascular amyloid consists mostly of fibrillar forms of Aβ, which stain with methoxy-X04, and not of oligomeric Aβ. In conjunction with the reduced effector function of crenezumab on microglial activation, lack of vascular Aβ binding in these preclinical models [13–15, 17] may help explain the reduced occurrence of ARIA and ARIA-E observed in the clinical trials of crenezumab [11, 17, 22].

Exploratory post hoc analyses of phase II clinical trials in mild to moderate AD patients showed that the higher of two crenezumab doses tested (i.e., i.v. infusions of 15 mg/kg every 4 weeks vs subcutaneous injections of 300 mg every 2 weeks) reduced cognitive decline in the milder subset of patients (Mini-Mental State Exam 22–26) [17, 22], suggesting that earlier treatment and greater brain exposure of crenezumab may provide clinical benefit. In addition, a recent publication [60] showed that crenezumab was able to significantly reduce oligomeric Aβ levels measured in the CSF from this same patient population. However, pivotal phase III trials (CREAD, CREAD2) in prodromal to mild AD patients were recently discontinued following a pre-planned interim analysis in CREAD that indicated crenezumab was unlikely to meet the primary endpoint, even though patients were given four times the phase II dose of crenezumab (60 mg/kg, i.v. every 4 weeks). Biomarker analyses of the phase III studies are ongoing, and thus, engagement of oligomeric Aβ and its relation to cognitive improvement is yet to be confirmed.

## Conclusions

Crenezumab’s selective binding to oligomeric versus monomeric Aβ species, both in vitro and in vivo, is a key component of crenezumab’s mechanism of action and has major implications for its differentiation from other therapeutic anti-Aβ monoclonal antibodies. In vivo localization of crenezumab binding in PS2APP mice to regions hypothesized to be rich in oligomeric Aβ, including the halo around amyloid plaques, dystrophic neurites, and hippocampal mossy fibers, suggests that crenezumab may interfere with oligomeric-mediated pathogenic signaling by engaging and possibly neutralizing oligomeric forms of Aβ. The absence of crenezumab binding to the dense core of plaques and to vascular amyloid, together with the reduced effector function of its IgG4 backbone, is consistent with a lack of plaque removal or increased incidence of ARIA-E in patients treated with crenezumab.

## Data Availability

Data sharing is not applicable to this article as no datasets were generated or analyzed during the current study.

## Abbreviations

Aβ: Amyloid β
Aβ_40_: Amyloid β 1–40 peptide
Aβ_42_: Amyloid β 1–42 peptide
AD: Alzheimer’s disease
ANOVA: Analysis of variance
APP: Amyloid precursor protein
ARIA: Amyloid-related imaging abnormalities
ARIA-E: Amyloid-related imaging abnormalities representing vasogenic edema
AUC_last_: Area under the curve from the time of last dosing to the last measurable concentration
BACE1: Beta-secretase 1
BSA: Bovine serum albumin
CHAPS: 3-[(3-Cholamidopropyl)dimethylammonio]-1-propanesulfonate hydrate
CL_obs_: Total body clearance
*C*_max_: Maximum concentration
CSF: Cerebrospinal fluid
dAβ: Dimeric amyloid β
EDTA: Ethylenediaminetetraacetic acid
ELISA: Enzyme-linked immunosorbent assay
FcγRs: Fcγ receptors
GFP: Green fluorescent protein
hAPP: Human amyloid precursor protein
hIgG: Humanized immunoglobulin G
hIgG4: Humanized immunoglobulin isotype G4
HMW: High molecular weight
Ig: Immunoglobulin
IntDen: Integrated density
i.p.: Intraperitoneal
i.v.: Intravenous
KO: Knockout
LAMP1: Lysosomal-associated membrane protein 1
mAb: Monoclonal antibody
moAβ: Monomeric amyloid β
NaCl: Sodium chloride
Ntg: Nontransgenic
PBS: Phosphate-buffered saline
PBST: PBS-Triton X100
PK: Pharmacokinetics
sAPPα: Soluble alpha APP
SDS-PAGE: Sodium dodecyl sulfate–polyacrylamide gel electrophoresis
tAβ: Trimeric amyloid β
TBS: Tris-buffered saline
WT: Wild type
